# Aquatic Exercise Is Effective in Improving Exercise Performance in Patients with Heart Failure and Type 2 Diabetes Mellitus

**DOI:** 10.1155/2012/349209

**Published:** 2012-04-23

**Authors:** Cider Åsa, Schaufelberger Maria, Stibrant Sunnerhagen Katharina, Andersson Bert

**Affiliations:** ^1^Institute of Neuroscience and Physiology, Sahlgrenska University Hospital, The Sahlgrenska Academy, University of Gothenburg, Mailbox 455, 405 35 Gothenburg, Sweden; ^2^Physiotherapy and Occupational Therapy Department, Sahlgrenska University Hospital, 413 45 Gothenburg, Sweden; ^3^Institute of Medicine, The Sahlgrenska Academy, University of Gothenburg, Mailbox 100, 405 30 Gothenburg, Sweden

## Abstract

*Background*. Peak oxygen uptake (VO_2peak_) and muscle function are more decreased in patients with a combination of chronic heart failure (CHF) and type 2 diabetes mellitus (2DM) compared to patients with only one of the conditions. Further, patients with 2DM have peripheral complications that hamper many types of conventional exercises. *Aim*. To evaluate the efficacy and applicability of eight-week aquatic exercise in patients with the combination of CHF and 2DM. *Methods*. Twenty patients (four women) with both CHF and 2DM (age 67.4 ± 7.1, NYHA II-III) were randomly assigned to either aquatic exercise or a control group. The patients exercised for 45 minutes 3 times/week in 33–34°C, swimming pool. *Results*. The training programme was well tolerated. Work rate (+11.7 ± 6.6 versus −6.4 ± 8.1 watt, *P* < 0.001) and VO_2peak_ (+2.1 ± 0.8 versus −0.9 ± 1.4 mL·kg^−1^·min^−1^, *P* < 0.001) and walking capacity (*P* = 0.01) increased significantly in the training group. Muscle function was also significantly improved and Hba1c decreased significantly (*P* < 0.01) during training, while fasting glucose, insulin, c-peptide, and lipids were unchanged . Training also increased vitality measured by SF-36 significantly (*P* = 0.05). *Conclusion*. Aquatic exercise could be used to improve exercise capacity and muscle function in patients with the combination of CHF and 2DM.

## 1. Introduction

Up to 25% of patients with chronic heart failure (CHF) have type 2 diabetes mellitus (2DM) [1], and it could be foreseen that this combination will be increasingly common [2]. 2DM entails a markedly increased risk of developing cardiovascular diseases [3]. Further, in all types of cardiovascular disease patients with 2DM have a significantly higher rate of mortality and morbidity than patients without [4]. On the contrary, CHF results in an insulin resistance syndrome which in turn could lead to the development of 2DM [5]. Both patients with CHF and 2DM, separately or in combination, suffer from reduced physical function, with decreased oxygen uptake and poor muscle function [6, 7]. In both conditions, similar pathological consequences are found in the skeletal muscle, such as an increased number of type II muscle fibres, low capillary density, and decreased oxidative capacity [8, 9]. Other similarities are signs of impaired endothelial function, which might be important to muscle function and physical performance [10, 11]. However, the impaired endothelial function might be corrected by exercise training [12]. The diseases do often impact negatively on activity of daily living and quality of life. These patients risk in a greater extent to develop depression and anxiety in comparison with a healthy population [13, 14]. There are consistent reports of improvement in physical performance and psychological function after aerobic and/or resistance exercise training in CHF as well as in 2DM [15, 16]. Since the prevalence of both conditions increases with age it is not unusual that patients also have other disabilities that further restrain physical ability. Aquatic exercise, that is, physical training in warm water, is an alternative exercise regimen, and we have recently shown positive effects of aquatic exercise in patients with CHF without 2DM [17]. To confirm the results of our previous study using aquatic exercise and to assess the efficacy of such training in patients with the combination of CHF and 2DM, this study was performed.

The hypothesis was that training in warm water would be safe and result in improvement in physical performance, muscle function, and metabolic control in patients with CHF and 2DM.

Therefore the aim of this study was to investigate the effect of aquatic exercise in patients with CHF and 2DM.

## 2. Methods

### 2.1. Patients

**Figure 1:**
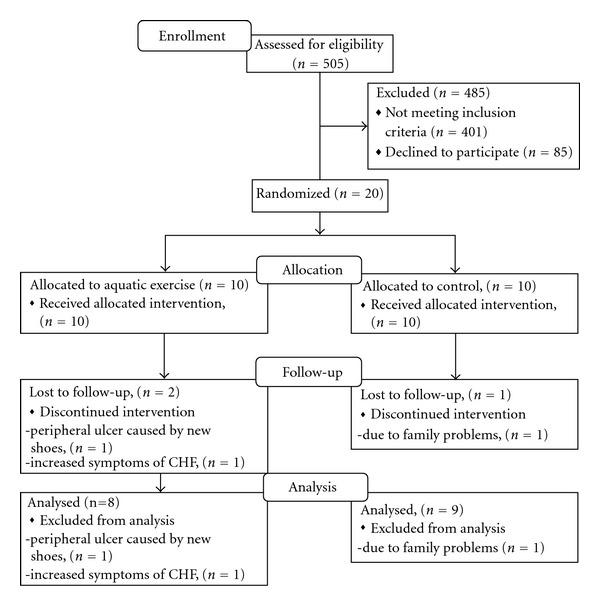
The inclusion process of patients.

Twenty patients (four women) with stable CHF and 2DM in NYHA class II or III, ejection fraction (EF) <50%, age above 55 years were included. Heart failure medication had to be stable for the previous three months. Exclusion criteria were peripheral artery disease, chronic pulmonary disease, status after stroke, or other disabling diseases that might interfere with the exercise protocol. The process of patient recruitment is described in Figure 1. After baseline testing, patients were randomised, using a 1 : 1 ratio, in a stratified according to order to 8 weeks of aquatic exercise (*n* = 10), or to a control period (*n* = 10). The patients were stratified according to age, NYHA class and gender. Baseline characteristics of the study population are given in Table 1. The study complied with the Declaration of Helsinki. The Ethics committee of Gothenburg University approved the research protocol, and informed consent was obtained from each subject. The testing procedures were repeated after eight weeks of training or control period, respectively.

### 2.2. Procedure

All patients performed the below given tests within 10 days before the randomisation and then during the last 10 days of the study period. Patients started on the first day with venous blood samples followed by an acquaintance test on the ergospirometer. Thereafter, questionnaires were filled out and the six-minute walk test performed. Finally patients performed on day 4–6 the maximal test on the ergospirometer and on day 8–10 the muscle tests.

### 2.3. Assessments

#### 2.3.1. Exercise Capacity

Work rate and peak oxygen uptake (VO_2peak_) were measured on an ergometer, using a ramp protocol with a 10-watt increase every minute until exhaustion. Expired gas was measured breath by breath using a *V*-max system (Sensor Medics, USA) as previously described [17].

#### 2.3.2. Six-Minute Walking Test

A standardised six-minute walking test was used to assess exercise capacity related to activities of daily living. The patients were asked to walk as far as possible during six minutes on a premarked 30-meter walkway [17, 18].

#### 2.3.3. Muscle Strength and Endurance

For measurement of isometric and isotonic strength and isotonic endurance the Biodex III (Biodex medical systems, New York, USA) was used. The test was preceded by a 5-minute warmup on a test bicycle. The subjects sat with a hip angle of 90°, and the right leg was attached to the lever arm of the dynamometer. Isometric knee extension strength was measured at a 60° knee angle. Isokinetic concentric strength was measured at 60°/s and at 180°/s for knee extensors. Isokinetic endurance was evaluated as the reduction of torque (in percent) between the first and the last three extensions in a series of 50 maximal contractions with an angle of 180°/s. Handgrip strength, the maximum grip force, and the mean value of the 10-second sustained grip was assessed by Grippit (AB Detector, Göteborg, Sweden). Clinical endurance tests, that is, unilateral isotonic heel-lift, bilateral isometric shoulder abduction and unilateral isotonic shoulder flexion were also measured. The test procedures have been described previously [17].

#### 2.3.4. Quality of Life

Health-related quality of life was measured using the Medical Outcome Short Form—36 (SF-36) [19] and disease-specific quality of life with the Minnesota living with heart failure questionnaire (LHFQ) [20]. Hospital anxiety and depression scale (HAD) was used to assess the level of anxiety and depression [21].

#### 2.3.5. Metabolic Function

Venous blood samples for assessing plasma glucose, HbA1c, serum insulin, serum C-peptide, and serum lipids were taken before and after the intervention period after an overnight fast and analysed according to the European Accreditation system [22].

#### 2.3.6. Training Programme

The training programme comprised 45-minute sessions in a heated pool (33°-34°C), three times a week over an eight-week period. The patients trained as a group following a low-to-moderate exercise level, that is, 40 to 75% of maximal heart rate reserve (HRR). The basis posture was standing with water just below neck level. The programme focused on peripheral muscle training but central circulatory exercises were also included as earlier described [17]. The control group was instructed to live their life as normal for eight weeks and was not allowed to increase their habitual physical activity during this period.

#### 2.3.7. Statistics

The SPSS 12.0 for Windows (Chicago, IL, USA) was used to analyse the data.

Ratio and interval data are given as mean (±1 SD or 95% CI) and ordinal data as median and range. Wilcoxon-matched pairs signed rank sum test was used for comparisons of paired observations within each study group. The Mann Whitney *U*-test was used to assess differences between groups. Nominal data between groups was compared by Chi-squared test or Fisher's exact test. A *P* value ≤ 0.05 was considered significant. A per-protocol design was used on all data.

## 3. Results

Aquatic exercise was well tolerated by the patients and no adverse events occurred during the aquatic exercise. Two patients in the training group were withdrawn, due to a peripheral ulcer caused by new shoes, increased symptoms of CHF, respectively. One patient in the control group was withdrawn, due to family problems. The average adherence (total number of attended sessions) was 92%. HRR during training ranged between 40% and 60% during peripheral muscle training exercises and between 55% and 75% during the aerobic exercises. In the training group two patients needed to reduce their insulin and one to take away the oral antidiabetics due to hypoglycaemia.

### 3.1. Exercise Capacity and Muscle Function

**Figure 2:**
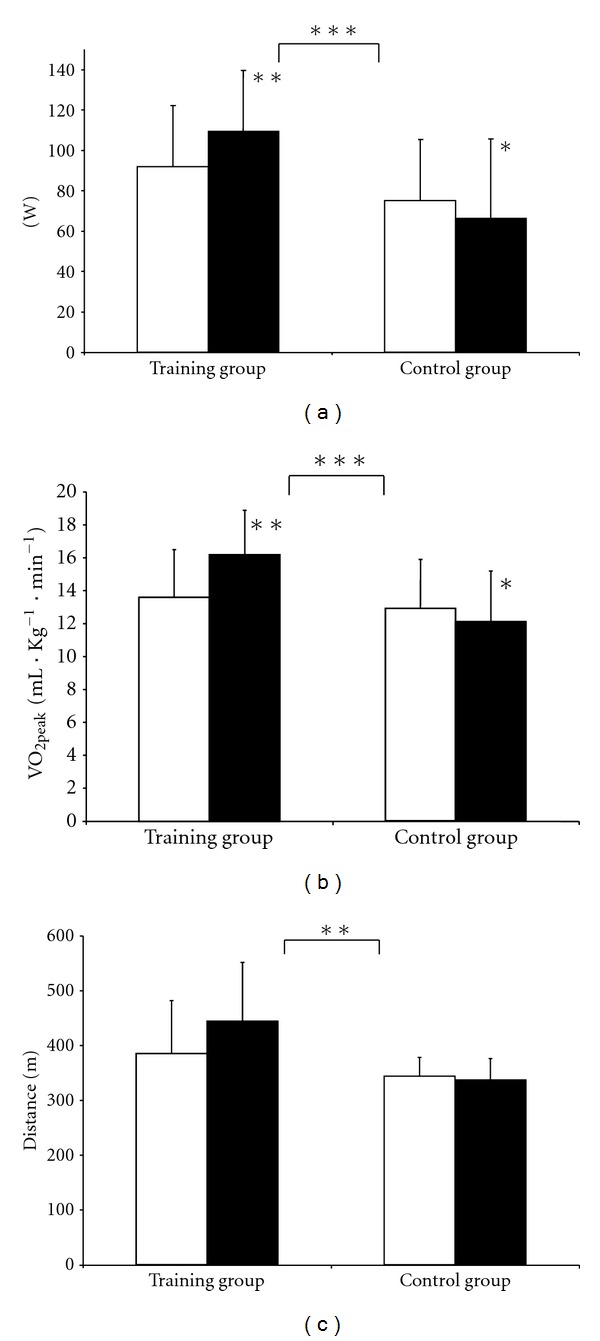
Work rate (a), peak oxygen uptake VO_2peak_ (b), and distance walked (c) in six minute walk test before □ (*n* = 10 and 10) and after ■ (*n* = 8 and 9) eight weeks of aquatic exercise.

Physical performance was significantly improved in the training group compared with the control group, regarding work rate, VO_2peak_ (*P* < 0.001), and walking capacity (*P* = 0.01) (Figure 2(a)–2(c)). There were no significant differences in knee extension regarding isometric strength, isotonic strength 60°/s, or isotonic endurance, neither in handgrip strength or endurance. However, a significant increase in isokinetic strength 180°/s (*P* < 0.001), isotonic heel lift (*P* = 0.01), shoulder flexion (*P* < 0.05), and isometric shoulder abduction (*P* < 0.001) was found in the training group after aquatic exercise (Table 2).

### 3.2. Metabolic Function

Hba1c decreased during aquatic exercise, but there was no significant improvement in fasting plasma glucose, insulin, c-peptide, or blood lipids after eight weeks of training (Table 3).

### 3.3. Health Related Quality of Life

**Figure 3:**
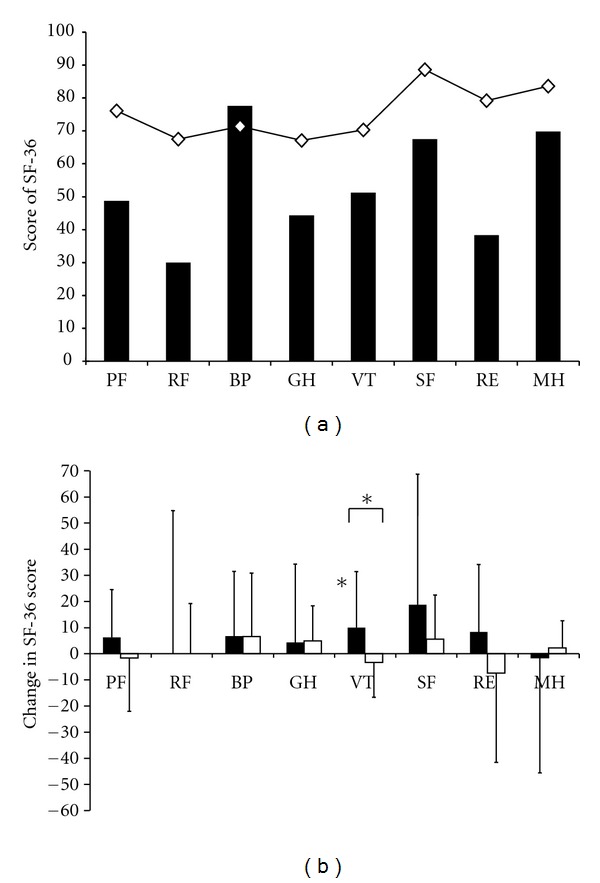
(a) Scores of SF-36 in all patients with chronic heart failure and type 2 diabetes mellitus, ■, (*n* = 20) compared to a Swedish healthy reference population -*◊*-. (b) Change in SF-36 after aquatic exercise, training group, ■, (*n* = 8) control group, □, (*n* = 9)*, *P* = 0.05.

Compared to a Swedish reference population [23], our patients with CHF and 2DM had lower SF-36 scores in all domains except for bodily pain, Figure 3(a). There was a significant difference in vitality scoring after aquatic exercise, whereas other domains were unchanged, Figure 3(b). Disease specific quality of life and grade of anxiety was unchanged in both groups after the intervention period, Table 4.

## 4. Discussion

This is the first study to show that aquatic exercise could be used as an effective tool to improve physical function in patients with the combination of CHF and 2DM. Further, the study confirms the results of our previous study with warm water training in elderly patients, supporting that this training is safe for patients with CHF.

### 4.1. Exercise Performance

A number of studies have demonstrated that exercise training on land, aerobic and resistance exercise improve function in patients with either CHF or with 2DM [15, 16]. VO_2peak_ is an important prognostic marker in these patients [24, 25] and it is significantly more reduced in patients with a combination of 2DM and CHF, than in patients with only one of the diseases [26]. It was therefore an important finding in this study that aquatic exercise was associated with a significant improvement in VO_2peak_. Improved general performance was also shown as an increase in work rate and walking capacity. The physiological reason for this improvement was not investigated, but others have shown that exercise in the two diseases separately results in cardiac and peripheral muscle function enhancement which improves cardiac output and arteriovenous oxygen difference. Elevated peripheral resistance and poor endothelial function are factors that might contribute to reduced exercise capacity; however it could be enhanced by physical training [15, 16]. Immersion in warm water results in immediate improvement in cardiac function, probably mediated by peripheral vasodilation and unloading of left ventricular function [27, 28]. Whether such effects would be more beneficial during long-term treatment, compared with training on land, would need further studies comparing the two exercise regimens.

### 4.2. Muscular Performance

Isokinetic strength in knee extensors was merely significantly improved at 180°/s and not in isokinetic strength at 60°/s or in isokinetic endurance and isometric strength. We have previously been hypothesised that an absent improvement in knee muscle function during aquatic exercise is due to the difficulty to gain enough resistance for this large muscle group in water [17]. However, the sensitivity of the test does also have large impact of the test results after training. Studies have shown that isotonic knee extensor training did not result in isokinetic knee extensor improvement [29, 30]. The increment in knee extension at 180°/s might be due to an enhanced neural adaptation since this test mirrors the ability to develop power [31, 32]. It seems less likely that this improvement should be attributable to an increase in the amount of type II fibres, after a relatively short endurance training of eight weeks. Aquatic exercise resulted also in increased isometric and isotonic muscle endurance measured by clinical endurance tests. These tests were performed exactly the way as it was trained. Specific adaptations in skeletal muscle after exercise seem to benefit patients with 2DM since the active muscle tissue reveals a higher metabolic rate in glucose metabolism [15]. An important finding in this study was that the training maintained and improved endurance in both upper and lower body muscle groups, which is important for older people to prevent falls and to accomplish daily tasks of living requiring both static and dynamic efforts [33].

### 4.3. Metabolic Control

No specific advice concerning diet or diabetic treatment was given during this study. Diabetic therapy was supplied by the patient's ordinary health care and was not part of the study. A positive finding was the decrease in HbA1c after training. However, other markers of metabolic control did not change. It was not the scope of this study to investigate insulin resistance, and others have shown signs of decreased insulin resistance after exercise in 2DM [34] however, the effect of training is unclear in CHF [35]. We could not confirm that immersion in warm water solely could enhance metabolic function in patients with 2DM, as shown by Hooper [36].

### 4.4. Quality of Life Measurements

The size of the population in this study was inadequate to show unequivocal changes in quality of life. Of the instruments used, only an index in SF-36, vitality increased after aquatic exercise. Since the level of anxiety and depression was low among most of our patients at baseline no effect was seen in HAD scores.

### 4.5. Aquatic Exercise

Aquatic exercise enables a combination of aerobic and resistance exercises and is especially suitable for patients with advanced age, obesity, peripheral neuropathy, orthopaedic problems, or other comorbidity that hampers exercises on land. Due to the buoyancy effect in water weight bearing activities are much more effortless to perform in water [37]. For example, it is more uncomplicated for a patient with peripheral neuropathy to walk in water.

The rate of adherence in this supervised short-term exercise study was high, which is in accordance with several other studies in patients with CHF [17, 38, 39] as well as with 2DM [24]. However, the long-term adherence in nonsupervised exercise has been reported low by others [40, 41]. A “smorgasbord” of physical training regimen to the patient's disposal might enhance the rate of adherence to prescribed exercise.

### 4.6. Limitations

Similar to many other exercise studies in patients with CHF, our study was performed in a limited number of patients which may restrict external validity. A marked difficulty was to recruit patients that were free from other disabling and complicating disorders like peripheral ulcers, infections, or problems with glycaemic control which are more common in patients with the combination of CHF and 2DM. Further, these patients have a higher morbidity that increases the risk of withdrawal during the study period. In clinical practice, these conditions might temporarily hinder participation in training programmes. However, a temporary stop in the programme should not exclude these patients from the beneficial effects of physical training in the long run.

## 5. Conclusion

Aquatic exercise is safe and effective to improve physical and metabolic function in patients with the combination of CHF and 2DM. Whether conventional exercise on land is equally effective has not been shown and would need further studies. Training in water is especially beneficial for those patients with other disabilities that obstruct exercises on land.

## Figures and Tables

**Table 1:** Demographic data of patients with chronic heart failure and type 2 diabetes mellitus.

Variables	Training (*n* = 10)	Control (*n* = 10)	*P* value
Age (years)	65.8 ± 5.8	69 ± 8.2	ns
Sex (F/M)	2/8	2/8	ns
Weight (kg)	93.6 ± 16.2	86.6 ± 24.2	ns
Height (cm)	176.1 ± 10	174 ± 8.8	ns
Duration of CHF (years)	5.3 ± 2.6	6.0 ± 5.2	ns
Duration of 2DM (years)	7.2 ± 5.8	6.9 ± 4.4	ns
LVEF (%)	34.1 ± 9.8	34.8 ± 9.1	ns
Etiology of CHF (IHD/DCM/HT)	8/1/1	4/4/2	ns
NYHA class (II/III)	5/5	3/7	ns
Chronic atrial fibrillation (*n*)	5	3	ns
Beta blockers (*n*)	9	8	ns
ACE-inhibitors (*n*)	8	9	ns
Diuretics (*n*)	9	9	ns
Digitalis (*n*)	2	5	ns
Insulin (*n*)	5	3	ns
Anti diabetics (*n*)	5	8	ns
Acetyl-salicylic acid (*n*)	5	8	ns
Warfarin (*n*)	5	3	ns

F/M: female and male, LVEF: left ventricular ejection fraction, NYHA: New York Heart Association classification, IHD: ischemic heart disease, DCM: dilated cardiomyopathy, HT: hypertension, ACE: angiotensin converting enzyme, *n*: number, ns: not significant.

**Table 2:** Muscle function before and after aquatic exercise in patients with chronic heart failure and type 2 diabetes mellitus.

Knee extension in Biodex III. Isokinetic		Before (*n* = 10/10)	After (*n* = 8/9)	*P* value within the group	*P* value versus the control group
Peak torque (60°s Nm) right leg	T	122 ± 41	127 ± 34	ns	ns
	C	102 ± 30	98 ± 32	ns	
Peak torque (180°s Nm) right leg	T	88 ± 28	119 ± 54	0.02	<0.001
	C	66 ± 22	64 ± 24	ns	
Endurance decline in %, left leg	T	46 ± 17	44 ± 13	ns	ns
	C	51 ± 14	52 ± 16	ns	
Isometric					
Peak torque	T	136 ± 41	136 ± 40	ns	ns
60° (N) right leg	C	109 ± 37	101 ± 32	ns	
Hand strength					
Peak force (N)	T	342 ± 121	385 ± 106	ns	ns
Right hand	C	248 ± 82	221 ± 62	0.04	
Peak force 10 s (N)	T	289 ± 108	323 ± 89	ns	ns
Right hand	C	207 ± 77	187 ± 62	ns	
Clinical endurance tests					
Heel lift (n.o)	T	14 ± 7	18 ± 6	0.01	0.01
	C	14 ± 4	14 ± 5	ns	
Shoulder flexion (n.o)	T	26 ± 11*	36 ± 12	0.02	0.03
	C	17 ± 8	17 ± 28	ns	
Shoulder abduction (s)	T	75 ± 25	89 ± 27	0.01	<0.001
	C	64 ± 26	56 ± 22	0.03	

T: Training group, C: control group, ns: not significant, n.o.: number of, *: *P* ≤ 0.05 at baseline between training and control group.

**Table 3:** Metabolic function before and after aquatic exercise in patients with chronic heart failure and type 2 diabetes mellitus.

Variables		Before (*n* = 10/10)	After (*n* = 8/9)	*P* value within the group	*P* value versus the control group
Hba1c (%)	T	7.9 ± 2.9	7.2 ± 0.9	0.01	ns
	C	6.9 ± 2.0	6.7 ± 3.2	ns	
P-Fasting glucos (mmol/L)	T	10.2 ± 2.9	9.3 ± 2.6	ns	ns
	C	7.8 ± 3.3	6.9 ± 2.0	ns	
S-Insulin (mU/L)	T	20 ± 5.7	20.1 ± 11.5	ns	ns
	C	19.2 ± 14.9	16.1 ± 14.0	ns	
S-C-peptide (nmol/L)	T	0.8 ± 0.4	1.1 ± 0.5	ns	ns
	C	1.0 ± 0.9	1.4 ± 1.5	ns	
S-Triglycerides (mmol/L)	T	2.4 ± 3.4	2.2 ± 2.1	ns	ns
	C	1.9 ± 1.0	1.6 ± 0.8	ns	
S-Cholesterol (mmol/L)	T	4.2 ± 1.0	4.3 ± 0.9	ns	ns
	C	4.2 ± 1.1	4.1 ± 0.5	ns	

P: plasma, S: serum, T: Training group, C: control group.

**Table 4:** No significant changes in disease specific quality of life and grade of anxiety and depression occurred after eight weeks of aquatic exercise.

LHFQ		Before (*n* = 10/10)	After (*n* = 8/9)	HAD	Before (*n* = 10/10)	After (*n* = 8/9)
Total score	T	48 ± 22	43 ± 15	Anxiety	5.1 ± 4.8	5.4 ± 3.7
	C	35 ± 13	35 ± 16		3.1 ± 1.8	3.5 ± 3.0
Physical dimension	T	22 ± 14	16 ± 8	Depression	3.5 ± 3.2	3.6 ± 2.3
	C	19 ± 11	20 ± 13		4.2 ± 2.7	4.9 ± 3.7
Emotional dimension	T	10 ± 8	7 ± 6
	C	5 ± 4	5 ± 4

LHFQ: Minnesota living with heart failure questionnaire HAD: hospital anxiety and depression scale. T: Training group, C: control group.
